# Comparison Adsorption of Cd (II) onto Lignin and Polysaccharide-Based Polymers

**DOI:** 10.3390/polym15183794

**Published:** 2023-09-17

**Authors:** Elena Ungureanu, Maria E. Fortună, Denis C. Țopa, Carmen O. Brezuleanu, Vlad I. Ungureanu, Ciprian Chiruță, Razvan Rotaru, Bogdan M. Tofanica, Valentin I. Popa, Doina C. Jităreanu

**Affiliations:** 1 “Ion Ionescu de la Brad” Iasi University of Life Sciences, 3 Mihail Sadoveanu Alley, 700490 Iasi, Romania; eungureanu@uaiasi.ro (E.U.); topadennis@uaiasi.ro (D.C.Ț.); olgutabrez@uaiasi.ro (C.O.B.); kyru@uaiasi.ro (C.C.); doinaj@uaiasi.ro (D.C.J.); 2 Institute of Macromolecular Chemistry “Petru Poni”, 41A Grigore Ghica Voda Alley, 700487 Iasi, Romania; rotaru.razvan@icmpp.ro; 3 Polytechnic University of Timisoara, 2 Piata Victoriei Alley, 300006 Timisoara, Romania; vlad.ungureanu@student.upt.ro; 4 “Gheorghe Asachi” Technical University of Iasi, 73 Prof. Dr. Docent Dimitrie Mangeron Blv., 700050 Iasi, Romania; vipopa@tuiasi.ro

**Keywords:** polysaccharide, biopolymer, lignin, adsorption, cadmium ion

## Abstract

Given the predominantly negative impact of heavy metals on living organisms, the present study proposed to evaluate the adsorption performances under static conditions of Cd (II) from aqueous solutions on unmodified Sarkanda grass lignin compared to the adsorption performances of polysaccharide polymers chemically functionalized, obtained by synthesis and in their native state, but which, although effective, have a cost price that does not allow for large-scale expansion. To improve the retention of Cd (II) on this aromatic component of the biomass resulting from the processing of lignocellulosic materials, different experimental conditions (pH, concentration, dose and contact time) were followed. The Freundlich and Langmuir isotherms were used to describe the equilibrium conditions. Adsorption kinetics were assessed using the Lagergren I and Ho and McKay II kinetic models, furnishing informative insights into the process mechanism. Lignin adsorption capacity was also analyzed by performing biological tests on tomato seeds (*Lypercosium esculentum*), since heavy metals are known to be a stress factor for seeds by disturbing the osmotic equilibrium. Through the prism of the investigated parameters and under precisely established experimental conditions, unmodified Sarkanda grass lignin—an aromatic biopolymer—can be recommended as a promising adsorbent for the retention of Cd (II) from aqueous solutions, successfully replacing polysaccharide, especially cellulose-based polymers.

## 1. Introduction

Society has become sensible to the harmful effects of pollutants such as heavy metals, xenobiotics, and waste based on synthetic polymers, which requires the development of phytoremediation processes that are effective, inexpensive, and ecologically non-disturbing and can be applied on a large scale. From point view of their ecological significance for the existing trophic chains, where they can interfere with the functioning of vital cellular components and persist in the long term [1], heavy metals present in the environment, whether from natural sources (igneous and sedimentary rocks, erosion, and formation processes of soils) or from anthropogenic sources (industrial activities, transport, and agriculture [2,3]) are considered threats to living systems [4]. The reduction in the number of beneficial microorganisms in the soil because of the high concentration of heavy metals can influence the decrease in the decomposition process of organic matter, leading to a reduction in soil fertility. The consequences of heavy metal stress include disturbances of metabolic processes, including seed germination disorders [5].

The mechanisms of accumulation of heavy metal have not yet been fully elucidated. Some heavy metals (Hg, Cd, Pb), have no physiological role and, moreover, have harmful effects on human health [6]. It is also possible that heavy metals effects on plants can lead to changes in proteins [7] or in the metabolism of carbohydrates and amino acids [8]. The result of changes in the processes of autoxidation of enzymes and peroxidation of lipids [9] is the consequence of an increased concentration of active oxygen [5]. By the impact of its toxicity on the ecological, evolutionary, nutritional, and environmental segments, cadmium can be considered a “main threat” to life because it is non-biodegradable, accumulates in the environment, and, subsequently, contaminates the food chain.

Due to their increased susceptibility to biodegradation, the waste of some natural polymers, such as lignin, the second most abundant organic macromolecule in plants after cellulose, is easily bio assimilated without the need for additional operations, aimed at facilitating the degradative action of environmental factors [10]. Porous or fibrous materials for the removal of saturated hydrocarbons, the heavy metals or oils work, according to two principles: adsorption (adsorbents) and absorption (absorbents) [11]. Usually porous and/or fibrous materials present both phenomena in contact with a liquid: absorption (by capillary action) and adsorption (by surface phenomena). In this case, the phenomenon is generically called sorption, and the sorbent material. Natural cellulosic materials such as lignin, moss, peat, sawdust, kapok (Java cotton), and kenafal (Deccan hemp or Java jute) are cheap, renewable, and abundant in the plant ecosystem [12,13]. There are literature data that attest to the quantitative retention of metal ions on lignin [14], but the adsorption capacity of heavy metal ions differs significantly depending on the origin of the lignin and the delignification process used.

The phenomenon of sorption of a heavy metal through the surface of a solid material occurs through physical attraction forces such as van der Waals, Debye, and Kiesom forces, components of energy dispersion of the London type [15]. Other studies show that the sorption phenomenon is closely related to the presence of hydrophobic groups on the surface of the sorbent material [16,17]. The important chemical groups that could be responsible for good sorption are the O-H, COO-H, and C=O groups [18,19]. According to some researchers [20], the adsorption of metal ions on lignin occurs through ion exchange mechanisms, but there are also literature studies [21] that assume that the adsorption of metal ions on lignin is the result of the combination of several mechanisms, such as ion exchange, adsorption, and complexation [22,23,24]. Sorption capacity depends not only on the nature of the sorbent but also on the type of polluting to be collected [25]. At the global level, lignin resulting in the manufacture of cellulose or from hydrolysis technologies can be harnessed as a result of its origin from renewable resources at a low price. [1]. “Low-cost” materials seem to be a lever for the future in terms of the retention of heavy metal ions, and lignin, this main aromatic component of biomass, can be considered an alternative raw material for fossil resources which can represent a plausible solution in some previous studies demonstrating the efficiency of this lignocellulosic waste in the adsorption of lead, zinc, and arsenic ions [26,27].

Also, lignin is a polyaromatic, porous and branched polymers, resistant and stable to the action of acids but sensitive to the action of oxidizing agents, consisting of thousands of phenylpropane monomer units, C3–C6 polymerized, with a high content of functional groups, which chemical bonds with metal ions, and provide to lignin cationic exchange capacity to be able the removal of polluting species, such as heavy metal ions [28,29]. Through the action of oxidants on the lignin fractions, it is possible to form fractions with properties similar to fulvinic and humic acids, which, in the presence of Me(H_2_O)^2+^ type aqua complexes, have the property of substituting the 2/4/6 molecules from the inner coordination sphere of water, forming complex combinations [24].

The initial pH, sorbent dose, the concentration, the contact time, and the temperature are very important factors in optimizing the adsorption process of polluting species [26,27]. To obtain information about the adsorption capacity of a given material under well-defined experimental conditions that can then be used for the design of a large-scale industrial wastewater treatment system, it is necessary to model the equilibrium data obtained experimentally [30]. Adsorption processes are evaluated with the adsorption isotherms obtained graphically, which show how much solute is retained by a certain adsorbent under well-defined experimental conditions. To interpret of experimentally obtained adsorption isotherms, a series of mathematical models are used, generically called models of adsorption isotherms (Langmuir and Freundlich models) [30,31,32]. From an experimental point of view, it is useful to know the adsorption kinetics and the time at which the solute is removed from the solution to be able to develop an effective experimental strategy [30,32].

In the kinetic modeling, it is considered that the driving force of adsorption is precisely the difference between the concentration of the solute in the solid phase (retained on the adsorbent) and its concentration in contact with the adsorbent. The kinetics of the adsorption process depend both on the actual retention process and on the diffusion stages that govern the transfer of the solute from the solution to the active centers on the surface of the adsorbent, providing particularly important information regarding its mechanism. Therefore, when choosing a kinetic model, the initial and final concentrations of the solute in the solution at different time intervals will be taken into account [33]. Although the adsorption process is influenced by temperature, pH of the initial solution, dosage, solute concentration, etc., the kinetic equations take into account only the influences of the noticeable parameters on the global speed [32]. The most frequently used kinetic models that try to approximate as faithfully as possible the existing mechanism in the case of the adsorption of pollutants from aqueous solutions are the Lagergren pseudo-I order and Ho and McKay pseudo-II order models [30,31,32,33].

Considering these aspects, the present study proposed to test the adsorption capacity in static conditions of unmodified Sarkanda grass lignin, used as a chemical substrate for an aqueous solution of Cd (II), considering the results obtained in other previous studies through which Sarkanda grass lignin is recommended as an effective adsorbent for Pb (II), Zn (II), and As (III) retention [26,27].

## 2. Materials and Methods

### 2.1. Materials

Chemical materials main: Unmodified Sarkanda grass lignin nanoparticles (Sarkanda Grass-100SA-140, with the properties: COOH, mmol/g: 3.3; Aromatic OH, mmol/g: 1.7; Chelating capacity (m_echiv_/100 g): 67.14; Average porosity: 74%; Particle diameter (µm): approx. 1), supplied by Granit Récherche Development S.A., Lausanne, Switzerland) and CdSO_4_•8H_2_O supplied by ChimReactiv S.R.L., Bucharest, Romania.

Biological material: Tomato seeds (*Lypercosium esculentum* variety) offered by “Ion Ionescu de la Brad”, Iasi University of Life Sciences, Iasi, Romania.

### 2.2. Experimental Procedure

**Scheme 1:**
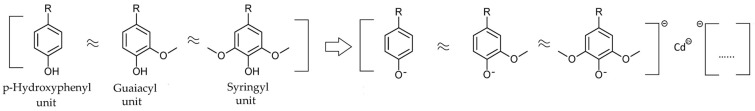
Potential initial interaction between the lignin structural units and Cd (II).

It was supposed that in contact with the polluting species, in the present case Cd (II), lignin, through its porous structure and the functional groups it has, will probably trigger an activated adsorption attributed to dissociation and interaction with the metal ion, forming complex combinations (Scheme 1).

**Scheme 2:**
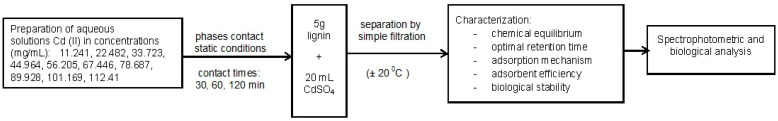
Experimental program.

The preparation of the stock solutions (0.001 mg/L) consisted of the dissolution of CdSO_4_·8H_2_O, in distilled water. The experimental program is shown in Scheme 2.

#### 2.2.1. Adsorption Experiments

In order to be able to develop an effective experimental strategy, it is necessary to accurately establish the ideal experimental conditions. To this purpose, the preceding experiments targeted the different conditions of concentrations of Cd (II), the pH, the temperature, the dosages, and the contact times. Experiments were performed at ±20 °C with 5 g of lignin in 1 L of Cd (II). The significant parameters that influence the sorption processes are the pH, the species, the solubility of metal ions, and the electric charge of the surface of the solid sorbent [19]. Literature studies recommend pH 6 for the adsorption of cadmium on different lignocellulose supports [31,34]. Following the experimental tests, the initial optimum pH was established at 6.2.

#### 2.2.2. The Germination Test

In order to have a definite conclusion on the efficiency of Cd (II) adsorption from aqueous solution on Sarkanda grass and on the toxic potential of the polluting species, biological tests were performed both on the contaminated lignin samples and on the filtrates resulting from the retention of the polluting species for tomato seeds of the variety *Lypercosium esculentum*, in laboratory conditions, in three repetitions. As control samples, distilled water was used for the filtrates and, for the contaminated lignin, uninfected adsorbent. To be able to estimate as faithfully as possible the impact of the polluting species on the evolution of the plants, the biological processes expressed by the energy and germination evolution, the height, and the mass of the seedlings were followed in order for a period of seven days.

### 2.3. Characterization Methods

#### 2.3.1. Surface Morphology

Scanning electron microscopy (SEM) was used (Brno, Czech Republic) using a Quanta 200 scanning electron microscope (5 kV) equipped with an EDX elemental analysis system (Ametek, Berwyn, PA, USA). Also, to capture the images in natural and polarized light, the trinocular polarizing microscope (Inf Plan 4/10/20/40: WF10x20: 20W Hal (TL) Kern OPM 181) was needed.

The wettability of Sarkanda grass lignin nanoparticles before and after sorption of Cd (II) from an aqueous solution was investigated by measuring the contact angle of a drop of liquid (water) placed on the surface of the sample using the KSV Cam 200 device from KSV Instruments Ltd. (Helsinki, Finland). Ten measurements were performed for each liquid guide drop.

#### 2.3.2. Spectrophotometric Determination of Cd (II)

For determining the concentration of Cd (II), Xylenol orange was used, with maximum absorption at 575 nm [31]. Quantitative determination of the metal ion obtained after filtration from the aqueous solutions was carried out by analysis of an exactly measured volume (2 mL) according to the experimental procedure, and the concentration value for each sample was calculated from the regression equation of the calibration curve.

For the spectrophotometric analysis, we used a Visible Spectrophotometer for laboratory, model VS-721N, 300-1000 nm, manufacturer JKI, Shanghai, China.

#### 2.3.3. Isotherm Models

Adsorption isotherms offer information about the distribution of solute particles between the two phases of the adsorption system [17,18,26,27]. The adsorption efficiency of Cd (II) was evaluated by Equation (1):

q = (c_i_ − c_e_)V/m, (mg/g) (1)

where c_i_—initial concentration (mg/mL), c_e_—equilibrium concentration (mg/L), V—volume of metal ion solution (L), m—mass of adsorbent (g).

The Langmuir isotherm is expressed using Equation (2):

q_e_ = q_max_(k_L_·c_e_/1+ k_L_·c_e_) (2)

where q_e_—amount of metal ions adsorbed per unit of mass of adsorbent (mg/g) at equilibrium, q_max_—maximum amount of metal ions retained on the absorbent after complete saturation (mg/g), K_L_—Langmuir constant related to the free energy of adsorption (L/mg), c_e_—equilibrium concentration of metal ions in solution (mg/L).

The Freundlich isotherm uses the empirical Equation (3), where, q_e_—amount of metal ions adsorbed per unit of mass of adsorbent (mg/g) at equilibrium, k_F_—Freundich constant, indicating adsorption capacity, n—constant characterizing the affinity of metal ions to sorbent, c_e_—concentration at equilibrium of metal ions in solution (mg/L) [26,27]:

q_e_ = k_F_·c_e_^1/n^ (3)

The linear regression analysis can be used to determine the most appropriate model that accurately represents the experimental data of the isotherm or the least squares method with which the correlation coefficient R^2^ is calculated [34].

#### 2.3.4. Kinetic Models

The kinetic parameters of Cd (II) adsorption on lignin are determined from the slopes and the intercept with the ordinate of the linear dependences lg (q_e_ − q_t_) and t, respectively, t/q_t_ and t, with the two commonly used Lagergren pseudo-I order and Ho and McKay pseudo-II order kinetic models. The Lagergren model for adsorption in a liquid–solid system and the Ho and McKay model are shown in Equations (4) and (5) [26,27,28]:

lg(q_e_ − q_t_) = lg q_e_ − (k_1_/2.303) t (4)

t/q_t_ = (1/k_2_·q_e_^2^) + t/q_e_ (5)

The choice of the most appropriate kinetic model for the verification of the experimental data was done with the help of linear regression.

#### 2.3.5. Biological Stability

To evaluate the seed germination parameters, the dimensions, and the mass of the resulting seedlings, germination tests were carried out for 7 days [35,36] in three repetitions with batches of 10 seeds each. The seeds were disinfected with NaClO 5% for 5 min, after which they were washed three times with MilliQ ultrapure water until the characteristic smell disappeared [37,38]. The seeds were placed in test tubes wide enough (180 × 18 mm) to allow their mixing with the filtrates and distilled water (control). Then, they were incubated for one hour with intermittent shaking to soak the seeds with filtrate or distilled water, according to the recommendations of ISTA (Seed Science and Technology, 1993) [39], after which they were evenly distributed in Petri dishes on filter paper (90 × 15 cm). The toxicity of filtrates and lignin contaminated with aqueous cadmium solutions was tested in the studied concentration domain at the three contact times of the phases: 30, 60, and 120 min. Distilled water was used as a control sample for filtrates, and uncontaminated lignin was used as a control sample for contaminated lignin.

The following were determined: the number of germinated seeds after three days (germination energy, E_g_, %) and the capacity of the seeds to germinate until the end of the germination period (germination faculty, F_g_, %) and the length and weight of the plant after seven days. E_g_ and F_g_ were determined with Equations (6) and (7) [32]:

E_g_ = (a/n)100 (6)

F_g_ = (b/n)100 (7)

where a—number of germinated seeds in the first third of the period, n—total number of analyzed seeds, b—number of germinated seeds at the end of the period (seven days of germination.

The seeds found swollen, rotten, and moldy at the end of the germination period were considered non-germinated. The experimental working parameters in the growth room (±24 °C, the alternation of 16 h light and 8 h dark) were chosen so as to match the specific living conditions of *Lypercosium esculentum*.

## 3. Results

### 3.1. Evaluation of the Efficiency Cd (II) Adsorption onto Sarkanda Grass Lignin

To evaluate the adsorptive performance of Sarkanda grass lignin, several experimental tests were carried out that had the main purpose of determining the stability of an optimum in terms of the pH, the concentration, dose, and the time of contact.

#### 3.1.1. Dose Lignin and Initial Concentration of Cd (II)

**Figure 1:**
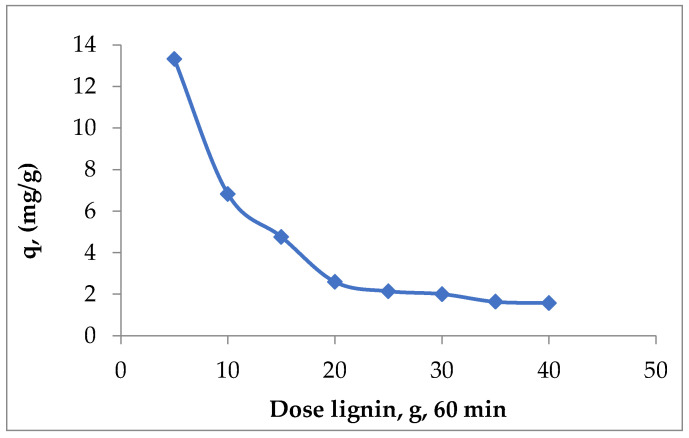
The influence of unmodified Sarkanda grass lignin dose on the efficiency of the Cd (II) adsorption in concentration 112.41 mg/L, contact time 60 min, pH 6.2.

The literature data recommend a dose of lignin fraction in the range of 4–40 g/L [33]. In this sense, experimental tests were carried out in the domain of 4–40 g lignin/L Cd (II) aqueous solution, and it was found that increasing the dose of lignin leads to a decrease in the quantity of metal ions retained per unit mass of adsorbent (Figure 1).

**Figure 2:**
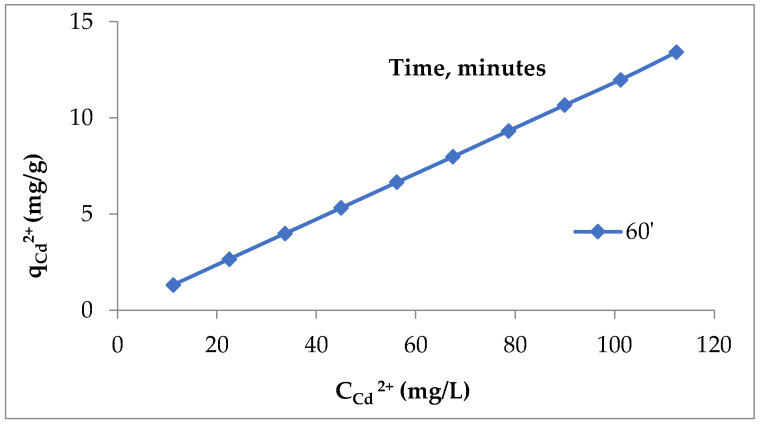
Quantity of Cd (II) retained per unit mass of unmodified Sarkanda grass lignin.

This variation of the values of q with the increase in the adsorbent dose can be explained as follows: with the increase in the adsorbent dose, the number of active centers (superficial functional groups) also increased; therefore, the amount of metal ions retained from the aqueous solution increased until reaching a maximum value. Further increasing the dose of adsorbent no longer had the same effect due to the phenomenon of agglomeration of lignin particles (a fact confirmed by microscopic analyses), which can lead to the blocking of superficial functional groups and, consequently, the values of q decreased. This is in accordance with the existing observations in the specialized literature, which show that, for a given adsorbent, the retention of metal ions from aqueous solutions occurs with high productivity when the experimental conditions are well selected [35,36]. Under these conditions and based on previous experiments [26,27], but also on the preliminary tests performed (Figure 1), it was estimated that a lignin dose of 5 g/L is optimal for the elimination of Cd (II) ions from aqueous solutions. To be able to estimate the adsorption efficiency, the quantity of Cd (II) that remains per unit mass of lignin (q, mg/g) was calculated. The increase in Cd (II) in the concentration domain studied determined a growth in the adsorption capacity of lignin from 1.3328 mg/g to the concentration of 11.241 mg/L to 13.4285 mg/g at the concentration of 112.41 mg/L, contact time 60 min (Figure 2).

When the concentration of Cd (II) in the solution increased, the quantity of cadmium retained on the lignin increased, a fact explained by increasing the ratio between the initial number of moles of Cd (II) and the number of accessible positions of sorption on the lignin fraction. Also, with the lignin pores being filled with the solution of the polluting species, no matter how much the concentration of the chemical species increases, adsorption became impossible, as the saturation state had been reached.

#### 3.1.2. Initial pH and Contact Time

**Figure 3:**
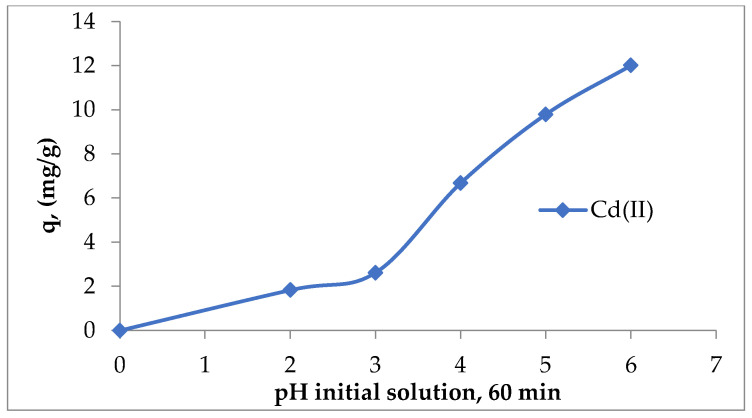
The influence of the pH of the initial solution on Cd (II) adsorption on unmodified Sarkanda grass lignin.

From the analysis of the speciation diagram of cadmium in aqueous solutions, it emerged that up to pH 10, the dominant species was Cd (II); after pH = 8.0, the dominant species was CdOH^−^ and Cd(OH)_2_ species begin to form, but the fractions of these species were lower than 30% for CdOH^−^ and 20% for Cd(OH)_2_, respectively [30]. Therefore, it is necessary to consider the role of H^+^ and OH^−^ ions in regulating the electric potential of the magnetic surface, which can lead to changes in the equilibrium characteristics of the adsorption process. At low pH, the excess of protons can compete with cadmium ions for the bonds with lignin, and the retained metal ions were lower. At high pH, due to the dissociation of functional groups from lignin, the level of retained metal ions on lignin was higher. On the other hand, at pH above 6.2, there was the possibility of Cd (II) precipitation in the form of hydroxide, which affects the adsorption efficiency [19]. Figure 3 shows the influence of the pH of the initial solution on Cd (II) adsorption on Sarkanda grass lignin (60 min contact time, ±20 °C).

As can be seen in Figure 3, the adsorption capacity of lignin for Cd (II) increased with an increase in the initial pH of the solution. This variation was mainly determined by the fact that with the increase in the pH of the initial solution, the deprotonation of the functional groups on the lignin surface (especially the carboxyl and hydroxyl ones) took place, which became negatively charged and can bind the positively charged metal ions from the aqueous solution. Regardless of the initial concentration of Cd (II) ions, at pH higher than 6.5, quantitative precipitation took place, with the lignin particles probably becoming precipitation centers for Cd (II) ions, which find on the surface of the adsorbent sufficient hydroxyl and carboxyl groups, in particular, to allow the transformation into a difficult soluble compound. Considering these aspects and following the experimental tests carried out (Figure 3), a pH of 6.2 was chosen.

**Figure 4:**
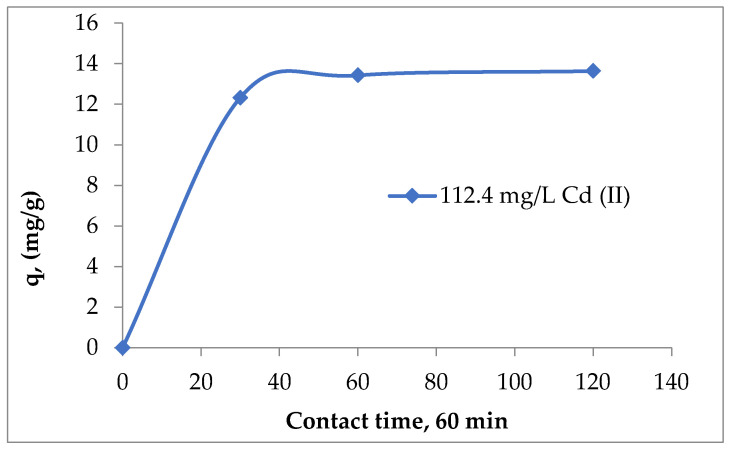
The influence of contact time on the adsorption of Cd (II) on unmodified Sarkanda grass lignin.

Considering the experimental data obtained (Figure 4) and the literature recommendations, which suggest a longer contact time between the phases because it can provide important clues about the interfacial dynamics and equilibrium [31,33], 60 min can be considered the optimal contact time for the absorption of Cd (II) on Sarkanda Grass lignin. Also, the contact time of 120 min was excluded because there were no variations in the adsorption capacity compared to those recorded at 60 min (Figure 4).

In other previous studies regarding the adsorption of Pb (II), Zn (II), and As (III) ions from aqueous solutions on lignin, the ideal contact time was also 60 min [26,27], which ensured good reproducibility of data and recommended this duration as being optimal for the adsorption of heavy metal ions from aqueous solutions on lignin Sarkanda grass substrate.

### 3.2. Assessment of Cd (II) Adsorption on Sarkanda Grass Lignin Based on Observations Deduced from Surface Analysis

**Figure 5:**
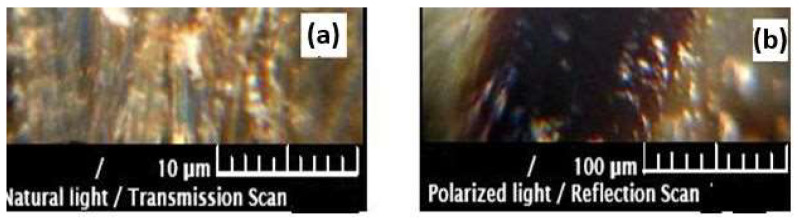
Microscopic images in natural (**a**) and polarized (**b**) light for unmodified Sarkanda grass lignin.

Microscopic studies show that lignin, through its porous structure (Figure 5), can act as a chemical substrate in which the functional groups are strongly linked to the skeleton and can retain polluting species.

**Figure 6:**
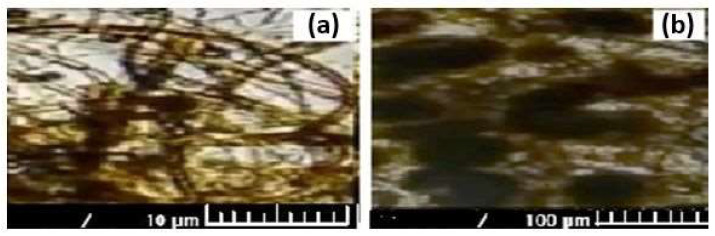
Microscopic images in natural (**a**) and polarized (**b**) light for Sarkanda grass lignin contaminated with Cd (II).

Microscopic images captured in natural (a) and polarized (b) light for the most contaminated Sarkanda grass lignin sample, at a concentration of 112.41 mg/L and contact time of 60 min (Figure 6), confirmed the diffusion of pollutant species in the pores of the adsorbent and the achievement of adsorption.

**Figure 7:**
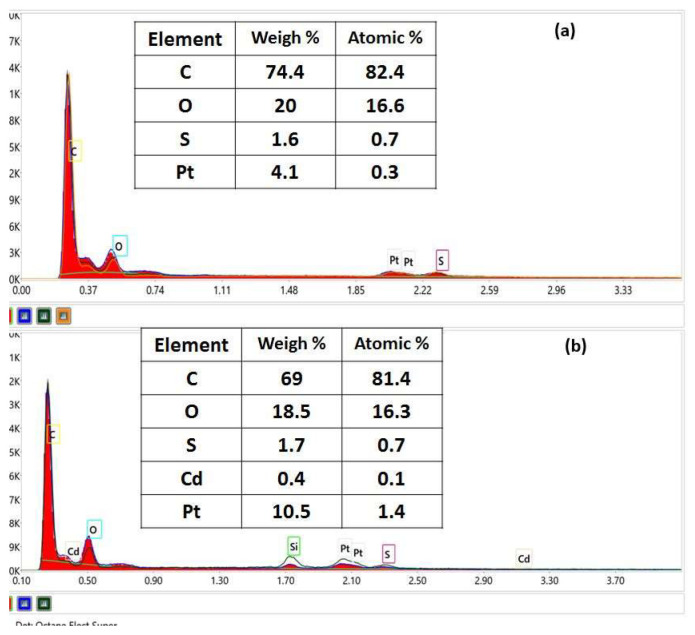
EDX elemental analysis for lignin before adsorption (**a**) and after Cd (II) adsorption (**b**).

**Figure 8:**
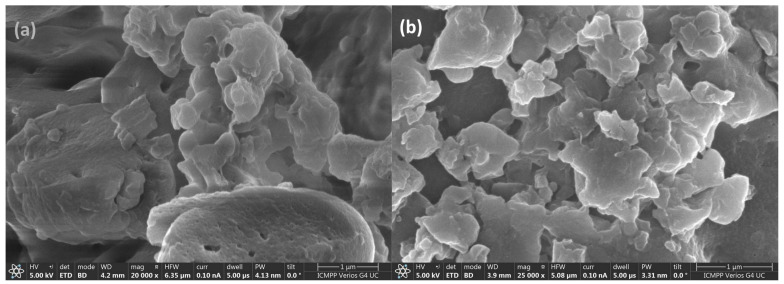
The SEM images for lignin before adsorption (**a**) and after Cd (II) adsorption (**b**).

The morphology and composition of the lignin before adsorption and after Cd (II) adsorption at a concentration of 112.41 mg/L and contact time of 60 min were identified by scanning electron microscopy (SEM) coupled with energy dispersive X-ray analysis (EDX) (Figure 7 and Figure 8).

The SEM micrograph of the unmodified lignin clearly showed well-separated particle agglomeration of about 4 μm lengths. The particles that form these agglomerations have micrometric dimensions, which confirm the diffraction analyzer.

The EDX spectra for the samples are given in Figure 7a,b, showing the main peaks of C, O, and S elements coming from both samples. The EDX spectrum for lignin after Cd (II) adsorption clearly shows the adsorption of cadmium. For SEM analysis, the prepared sample has been metallized with Pt to improve the contrast, thus resulting in Pt in both samples. EDX and SEM analyses of the unmodified lignin clearly show the contact, diffusion, and retention of the pollutant species in the lignin fraction in the pores of Sarkanda grass lignin particles.

### 3.3. Wettability Study

It is known that lignin contains, like any organic macromolecular compound, hydrophilic and hydrophobic groups that can influence the progress of sorption processes. The measured data are given in Table 1 for unmodified Sarkanda grass lignin (not introduced into the Cd^2+^ solution) and in Table 2 for the used lignin (after the sorption of the Cd^2+^ solution). Time represents the duration of the contact angle measurement; CA L and CA R represent the contact angle measured on the left and right of the drop; CA M represents the calculated average of the contact angle; L, H, and V represent the width, height, and volume of the water drop; and A represents the contact surface between the water drop and the lignin.

**Figure 9:**
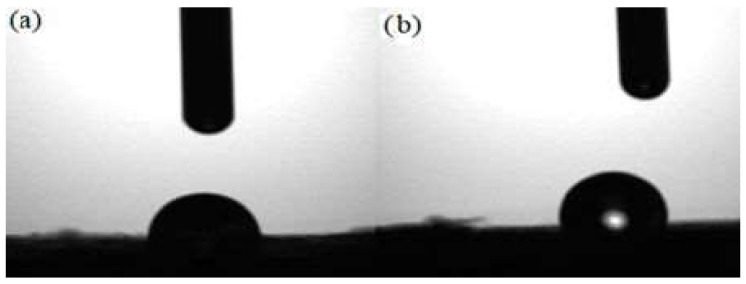
Contact angle images for unmodified lignin (**a**) and contaminated lignin (**b**) with Cd (II), in concentration 112.41 mg/L, contact time 60 min.

A contact angle with a value below 90 degrees means hydrophilic character (water absorbent), and a contact angle above 90 degrees means hydrophobic character. The unmodified Sarkanda grass lignin has a hydrophilic character, it absorbs water and implicitly also the aqueous solution of Cd (II), as a consequence of its porous structure. Contaminated Sarkanda grass lignin has a hydrophobic character, it repels water in this block, meaning that the lignin has absorbed the Cd (II) solution and reached saturation (Figure 9).

### 3.4. Modeling of Adsorption Equilibrium of Cd (II) onto Sarkanda Grass Lignin by Obtaining Freundlich and Langmuir Isotherms

The classic Freundlich and Langmuir isotherms show information about the type of adsorption, monolayer or multilayer, and are frequently used to model adsorption isotherms. The choice of the most appropriate model was made on the basis of the correlation coefficients (R^2^), which are calculated from the linear representation of each individual model and allow the quantitative evaluation of the efficiency of the studied adsorption process.

**Figure 10:**
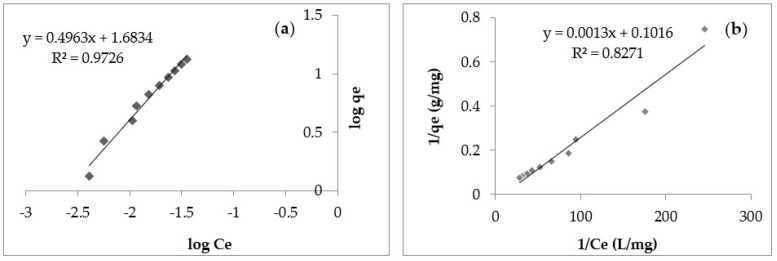
Freundlich adsorption model (**a**) and Langmuir adsorption model (**b**) for Cd (II) retention onto Sarkanda grass lignin after 60 min.

Figure 10 shows the Freundlich (a) and Langmuir (b) models for Cd (II) adsorption from aqueous solutions on Sarkanda grass lignin.

From Table 3, the values of the correlation coefficients (R^2^) obtained in the Langmuir model fell within the domain of 0.8219–0.8863 and were lower than the values of the correlation coefficients (R^2^) obtained in the case of the Freundlich model (0.9637–0.9810), showing that the experimental data better verified the Freundlich model.

By analyzing Table 3, it can be seen that the values of K_F_ fell within the domain 0.8321–1.0432, and those of 1/n were included in the domain 0.9041–0.9312, which indicates the presence of a binding energy by adsorption due to interactions of ion exchange or surface complexation. The values of K_L_ fell within the domain of 0.2457–0.3528, being lower, as can be seen, than the values of K_F_.

The value of 1/n was useful to intuit the nature and intensity of the interactions between the metal ions and the functional groups of the adsorbent. Thus, a value between 0 and 1 describes a favorable adsorption process. Moreover, the closer the value of 1/n is to 1, the stronger the interactions between the metal ions in the aqueous solution and the functional groups of the adsorbent. In the current study, the value of 1/n was in the range 0.9041–0.9312, which suggests favorable or active chemisorption with the formation of strong bonds.

Also, the contact time of 60 min seemed to be the optimal adsorption time. At 120 min, the changes were very small, a fact owed to saturation, when the concentration gradient and the adsorption speed decreased, which were otherwise normal behaviors in the case of surface processes such as adsorption.

### 3.5. Kinetic Modeling of the Adsorption of Cd (II) onto Sarkanda Grass Lignin

In order to interpret the kinetic data of the process, the experimental results were processed using Lagergren pseudo-I order and Ho and McKay pseudo-II order kinetic models. Through linear regression, using the Lagergren pseudo-first order and the Ho and McKay pseudo-II order kinetic models to reflect the adsorption capacity of the solid phase, the correlation coefficients (R^2^) were calculated.

**Figure 11:**
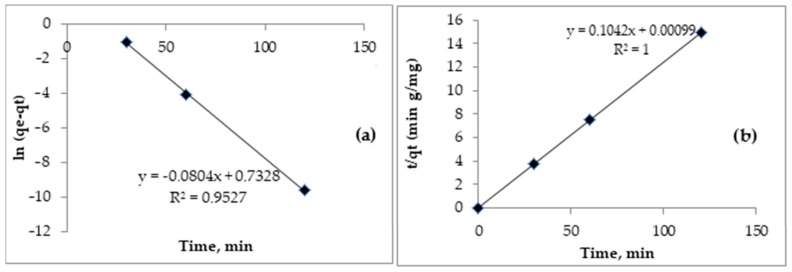
Lagergren pseudo-I order model (**a**) and Ho and McKay pseudo-II order model (**b**) for adsorption of Cd (II) onto Sarkanda grass lignin after 60 min.

In Table 4, the characteristic kinetic parameters are presented and calculated. The kinetic models are represented in Figure 11a,b.

For the Lagergren pseudo-I order kinetic model, the coefficients (R^2^) were lower than 0.9527, falling more precisely in the range 0.7108–0.9527 (Table 4), which indicates the appearance of electrostatic interactions between Cd (II) and the functional groups. On the lignin surface, chemisorption is favored at the expense of physical adsorption, which the Lagergren model cannot explain [16,17,18].

The experimental data were then processed using the Ho and McKay pseudo-II order kinetic model, and the concordance obtained was much better. In this case, the correlation coefficients (R^2^) showed unitary values in all situations, and the other parameters, q_e_ and K_2_, showed the good affinity of the tested chemical agents as pollutant species (Cd II) and as substrates chemically (Sarkanda grass lignin), suggesting an active adsorption attributed to the complexation capacity of the involved species, especially the availability of the functional groups of the lignin to associate covalently. Thus, when Cd (II) arrived near the lignin surface, it interacted with the functional groups on the lignin surface (R^2^ = 1), realizing an intense ion exchange that, thus, ensures the binding of cadmium to the lignin surface. Sarkanda grass lignin is presented as having a chelating capacity (m_echiv_./100 g) of 67.14, and the Ho and McKay pseudo-II order kinetic model, through the prism of the analyzed parameters, indicates the occurrence of chemical interactions (probably donor-acceptor) between the metal ion and the functional groups on the surface of the adsorbent and, implicitly, the complexation.

### 3.6. Evaluation of the Adsorptive Performances of Sarkanda Grass Lignin concerning Cd (II) Retention from Aqueous Solutions by Determining Some Biological Parameters

Testing the efficiency of Cd (II) absorption onto Sarkanda grass lignin can also be verified by evaluating some biological parameters, considering the negative impact of this chemical species on living systems.

#### 3.6.1. Number of Germinated Tomato Seeds, *Lypercosium esculentum* Variety

**Figure 12:**
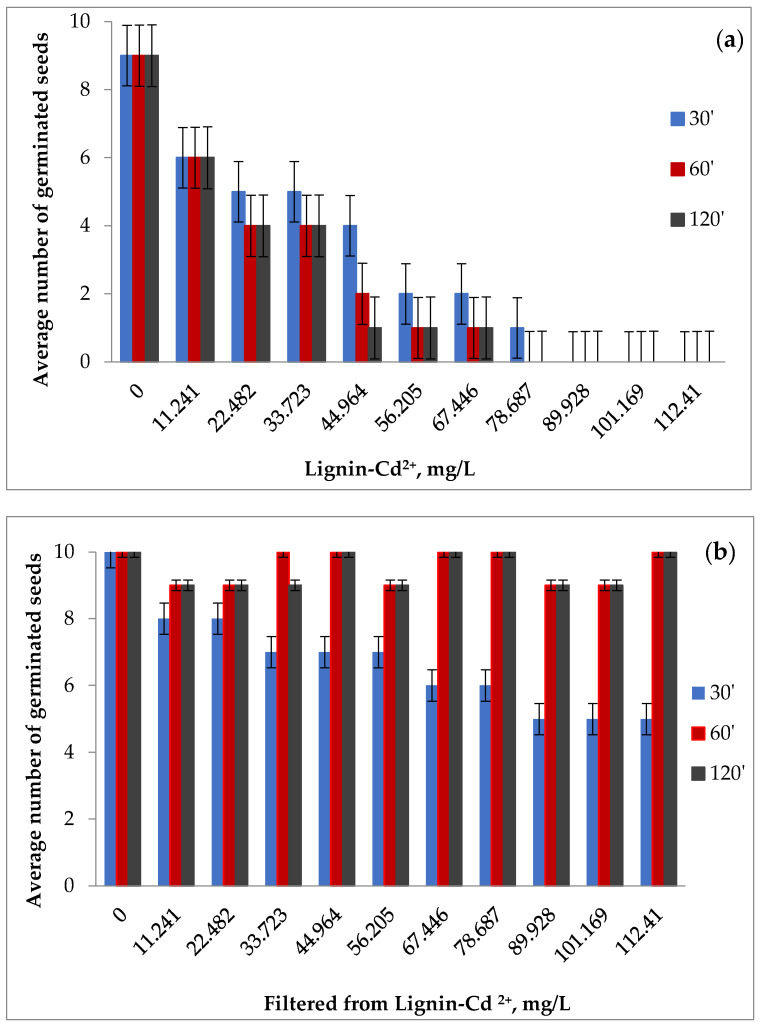

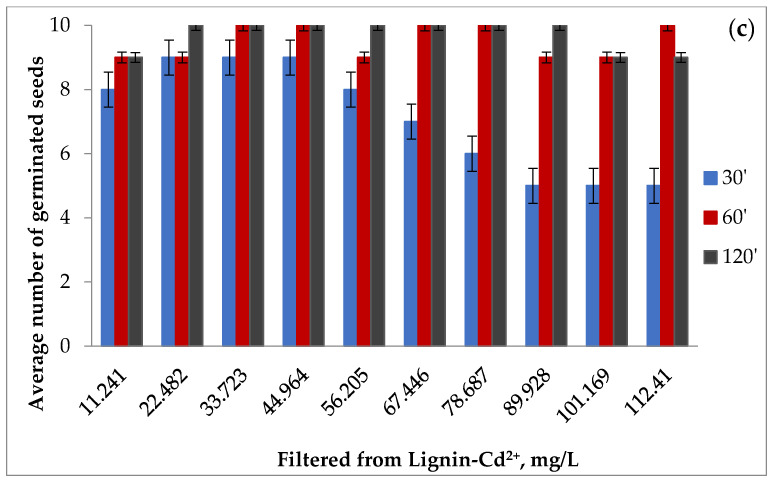
The average number of tomato seeds germinated at 3 days for the contaminated samples (**a**) and for the filtrates resulting from Cd (II) adsorption at 3 days (**b**) and 7 days (**c**).

Figure 12a–c shows the average number of germinated seeds at 3 days for the contaminated samples and the average number of germinated seeds for the filtrates resulting from Cd (II) adsorption at 3 days and at 7 days, for the three times of contact between phases.

The negative impact of cadmium on seed germination can be clearly observed, intensified by the growth of the concentration of metal ions and the increase in contact between the phases. In the reference samples, the number of germinated seeds was 9 for lignin and 10 for distilled water.

The germination of the seeds in the filtrate was interesting, where the number of germinated seeds after 3 days, but also the number of plants after 7 days after germination, were close to the ones obtained in the case of the control at the times of 60 and 120 min, but lower at the contact time of 30 min, which indicates the probability of a more attenuated adsorption of the polluting species in the first phase.

The data were consistent with the evolution of seed germination in the case of contaminated lignin samples. It was observed that at the contact time between phases of 30 min and at low concentrations in the polluting species, the highest number of germinated seeds appeared after 3 days, which showed a superficial retention of cadmium and confirmed the lower number of germinated seeds in the case filtrates at this contact time.

**Figure 13:**
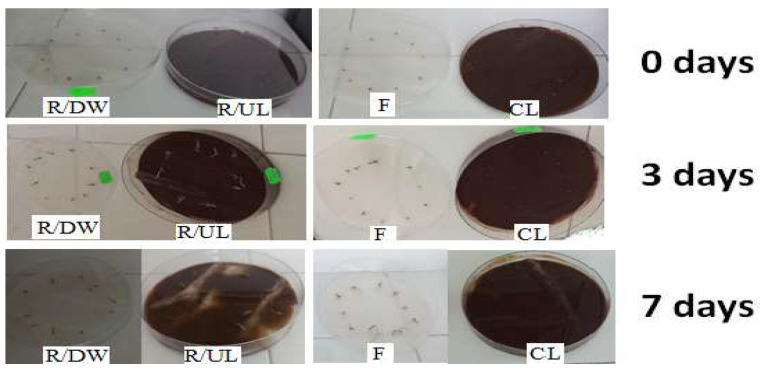
The germination of tomato seeds over a period of 7 days, at an adsorption time of 60 min and a concentration of 112.41 mg/L Cd (II).

Figure 13 shows the germination of tomato seeds for 7 days with reference/uncontaminated lignin (R/UL), lignin contaminated with cadmium (CL), reference/distilled water (R/DW), and the filtrate (F) obtained after adsorption for 60 min at a concentration of 112.41 mg/L Cd (II). 

#### 3.6.2. Germination Energy and Germination Faculty for Tomato Seeds

Table 5 shows that the germination energy was correlated with the number of germinated seeds for filtrates and for contaminated lignin samples.

At the contact time between phases of 30 min, the retention of cadmium was more attenuated, so that the filtrates were more concentrated and the germination energy was lower, but at 60 and 120 min, the germination energy was higher because the filtrates were more diluted as a result of the good retention capacity of lignin.

In the case of filtrates, the germination power varied proportionally with the germination energy, while for contaminated lignin, zero values were recorded in all situations.

#### 3.6.3. Mass and Average Height of Tomato Seedlings

**Figure 14:**
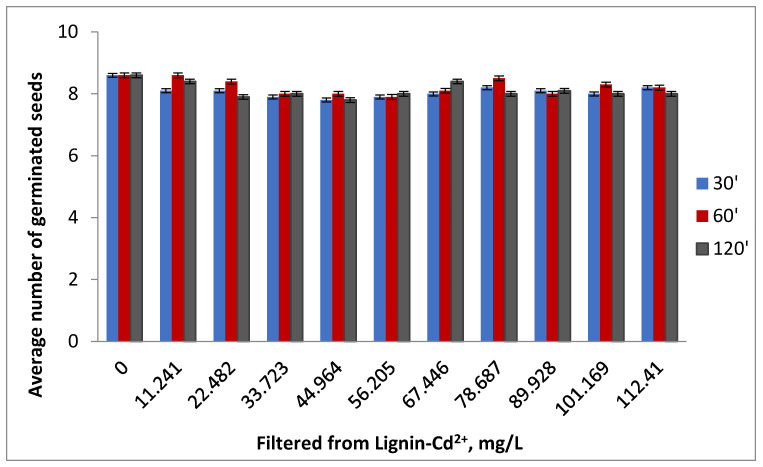
The mass and average height of tomato seedlings, developed in the filtrates resulting from Cd (II) adsorption on Sarkanda grass lignin.

Figure 14 shows that the mass and height of the seedlings developed in the case of the filtrates showed values close to those found in the reference, regardless of the contact times and the concentration of the retained pollutant species. In the case of contaminated lignin, these parameters could not be evaluated because, at the end of the germination, there were no seedlings, a fact attributed to the good adsorption capacity of lignin and implicitly to the toxicity of cadmium.

**Figure 15:**
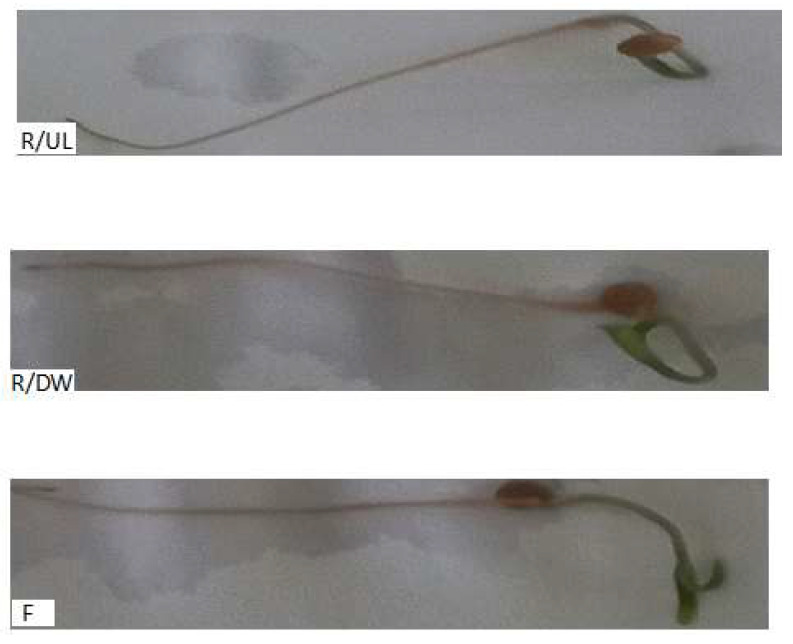
Tomato seedlings developed in reference and filtered samples 7 days after germination.

In Figure 15, tomato seedlings of the *Lypercosium esculentum* variety are presented, developed in laboratory conditions 7 days after germination for reference/uncontaminated lignin (R/UL), reference/distilled water (R/DW), and for filtrate (F) obtained after adsorption after 60 min at a concentration of 112.41 mg/L Cd (II).

Biological tests, by analyzing the evaluated parameters, demonstrated the efficiency of Sarkanda grass lignin in the adsorption of Cd (II) from aqueous solution, which seemed to be due to the ion exchange capacity of the involved species.

## 4. Discussion

### 4.1. The Influence of Lignin Sarkanda Grass Dose on the Efficiency of Cd (II) Adsorption from Aqueous Solution

Considering mainly the economic segment, in order to optimize the Cd (II) adsorption, the use of the smallest possible dose of lignin was pursued. The experimental data obtained recommend that the ideal dose of lignin from Sarkanda grass is 5 g/L Cd (II) aqueous solution (Figure 1). The increase in the concentration of polluting species contributes to a more pronounced adsorption on lignin until the adsorbent pores become inaccessible, a fact that can be explained if we consider that adsorption is a surface process. It seems that at saturation, most of the functional groups of lignin are occupied, so their diffusion towards the unreacted (free) functional groups, which are found inside the lignin particles, is difficult, a fact that respects the principle of mobile equilibrium. Also, the functional groups on the surface of lignin and its structural particularities can form coordinative bonds as a result of its good affinity with Cd (II).

### 4.2. The Effect of pH and Contact Time on the Adsorption of Cd (II) onto Sarkanda Grass Lignin

The initial pH influences the speciation and solubility polluting (whether they are ions metallic or organic dyes), and the capacity of separation of the functional groups on the surface of the adsorbent, considered adsorption centers mainly the economic segment, in order to optimize the experimental conditions of Cd (II) adsorption, the use of the smallest possible dose of lignin was pursued [33]. When the pH of the initial solution is greater than 8.0, most of the ions and metals from the solution precipitate in the form of hydroxides, and the adsorption process is either stopped or involves a totally different mechanism [19]. The results of the preliminary tests carried out regarding the retention of cadmium on Sarkanda Grass lignin as the adsorption substrate at pH variation recommend the value of 6.2 as being an optimal value (Figure 3). After a contact time of 120 min, the quantities of Cd (II) retained on Sarkanda grass lignin are identical, in most cases, to those recorded after a contact time of 60 min or vary negligibly (Figure 4). Although there are very small, almost imperceptible, variations in the amount of Cd (II) retained on Sarkanda grass lignin at times of 60 and 30 min, respectively, 60 min is recommended as the optimal contact time. The contact time of 60 min is also recommended from the perspective of surface and microscopic analyses and the contact angle, important parameters in the qualitative and quantitative characterization of surface processes such as adsorption, and agrees with the experimental results obtained in previous studies [26,27], where the adsorption of lead, zinc, and arsenium on Sarkanda grass lignin was tested under the same experimental conditions.

### 4.3. Highlighting the Adsorption of Cd (II) onto Sarkanda Grass Lignin through Surface Analysis

The presence of functional groups and the porous structure of lignin, demonstrated through microscopic studies (Figure 5), show that this material can be used as an adsorbent for the retention of polluting species from aqueous solutions and, therefore, can be utilized in environmental pollution reduction processes. Microscopic images (Figure 6) in natural (a) and polarized (b) light for Sarkanda grass lignin contaminated with Cd (II) reflect the possibility of the formation of chemical bonds (Sarkanda grass lignin-Cd (II)) and underline the cationic exchange capacity of this biomass fraction.

The composition of the lignin before and after adsorption of CdSO_4_ was obtained using EDX analysis. As expected, the surface morphology of the unmodified lignin was different from that observed in the lignin contaminated with Cd (II), a fact that confirms the diffusion of the polluting species and its retention in the pores of the lignocellulose substrate (Figure 8). The contact angle also analyzed and confirmed the interaction of lignin and metal ions. The unmodified lignin had values of the contact angle below 90 degrees (Table 2), which denotes the existence of some hydrophilic groups willing to participate in the ion exchange. In Table 3, the contact angle values recorded for lignin contaminated with Cd (II) exceeded 90 degrees, which corresponds to a hydrophobic character. Therefore, the lignin adsorbed the Cd (II) solution, saturation and hydrophobicity were reached, and the lignin pores became inaccessible (Figure 9).

### 4.4. Adsorption Isotherms

Modeling equilibrium data obtained experimentally in order to evaluate the adsorption capacity of a given material under exact experimental conditions allows for the acquisition of particularly useful information that can then be used to design a large-scale industrial wastewater treatment system. Studies in the literature [36,37] highlighted the fact that the more complex the composition of a wastewater, the more adsorption systems used for its treatment must be described and selected with greater accuracy. The experimentally obtained isotherms for the adsorption of Cd (II) on lignin in the studied concentration domain are non-linear, and their slope is typical. The literature recommends the use of the Freundlich model for the interpretation of adsorption processes that take place on heterogeneous surfaces or are composed of functional groups with different affinities for a given metal ion [37] and the Langmuir model to describe adsorption in monolayers and on homogeneous surfaces [20,24,28]. The values obtained for the Lagmuir K_L_ constant show that the adsorption would not take place in the monolayer, and the Sarkanda grass lignin surface is not perfectly homogeneous. Using the Freundlich constants (n and K_F_), the degree of adsorption can be estimated; thus, the nature of the process mechanism can be deduced. The higher the values of K_F_ and 1/n, the more intense the interactions between Cd (II) in the aqueous solution and the functional groups of lignin [26,27].

The experimental parameters obtained following the application of the Freundlich model have higher values than those obtained in the case of the application of the Langmuir model (Table 3), which would suggest that the Freundlich model is more appropriate to describe the retention of Cd (II) onto Sarkanda Grass. Analyzing the correlation coefficients (R^2^) obtained in the case of the two models (domains: 0.82–0.88 for the Langmuir model and 0.96–0.98 for the Freundlich model), the idea emerges that Sarkanda grass lignin seems to be an alternative as an adsorbent for Cd (II), but the physical or chemical nature of the adsorption cannot be established exactly, which makes the interpretation of the data by kinetic modeling necessary.

### 4.5. Kinetics of Cd (II) Adsorption onto Sarkanda Grass Lignin

The kinetics of Cd (II) adsorption on Sarkanda grass lignin were analyzed using the two models frequently recommended by the literature: Lagergren pseudo-I order and Ho and McKay pseudo-II order kinetic models [18,26,27,28]. After applying the two kinetic models through the prism of the parameters obtained (Table 4), it can be found that the adsorption of Cd (II) on Sarkanda grass lignin is more precisely described by the Ho and McKay pseudo-II order kinetic model, which shows the compatibility of the experimental data with this kinetic model. It is noteworthy that the correlation coefficient (R^2^) obtained at the Ho and McKay pseudo-II model was unitary in all case studies, which confirms the good adsorption capacity of Sarkanda grass lignin.

### 4.6. Verification of Cd (II) Adsorption Efficiency onto Sarkanda Grass Lignin by Biological Tests

It was based on the idea that in the case of low retention of the polluting species, the embryo of a seed would pass from the state of dormancy to the active state, and a seedling would appear—that is, the embryo would germinate. Therefore, the samples contaminated with the polluting species could clearly inhibit the germination process, given that a heavy metal, therefore also cadmium, represents a stress factor for seeds, being able to disturb the osmotic equilibrium or metabolic functions and replace essential nutrients through ion exchange [40]; hence, the indirect toxic effect on plants.

The experimental data (Figure 12) show that the presence of cadmium negatively influences the germination capacity of tomato seeds. Starting with the concentration of 33.723 mg/L, the number of germinated seeds was less than half of the total of 10, for all contact times, and for the concentration of 89.928 mg/L Cd (II), the number of germinated seeds was zero (the seeds are not germinated), which suggests both a more intense adsorption but also the negative effect of cadmium on the germination power of the seeds with the increase in concentration and duration of contact. Moreover, 7 days after germination, in the case of all samples of contaminated lignin, regardless of the contact time and concentration, no new germinated seeds appeared, and the existing seedlings died, which confirmed the toxicity of cadmium and its good capacity for lignin adsorption, as demonstrated by the kinetic and chemical equilibrium results obtained. It should be noted that seeds found swollen, rotten, or moldy at the end of the germination period were considered non-germinated.

As can be seen (Table 5), the germination energy and the germination power in the filtrates had values close to those in the control sample, regardless of the concentration of the polluting species that contaminated the lignin, which confirmed the efficient adsorption of Cd (II). Also, the values of these parameters at contact times of 60 and 120 min differed insignificantly, which agrees with the kinetic and chemical equilibrium conclusions, which give 60 min as the optimal adsorption time. The average mass and height of tomato seedlings developed in the case of the filtrates show values close to those found in the reference, regardless of the contact times or the concentration in Cd (II), a fact that confirms the good retention capacity of Sarkanda grass lignin. The mechanisms involved in the adsorption of metal ions on lignin are the subject of a continuous world debate that has several hypotheses. Therefore, further exploration of the complexation process and the nature of the active centers on the lignin substrate is a priority and a challenge that we want to develop in a future study.

We also considered studying the thermodynamic behavior of the adsorption of Cd (II) on Sarkanda grass lignin, because the thermodynamic parameters (variation of free energy, entropy, and enthalpy) can provide valuable information on the nature of interactions between chemical species involved in a process. The reproducibility of the experimental data, resulting from the approaches of chemical equilibrium, kinetic stability, and biological stability, recommends Sarkanda grass lignin, under precisely established experimental conditions, as a possible adsorbent, both from the point of view of its properties and from the point of view of the ratio price/quantity, given that it is one of the main fractions of biomass and implicitly a reusable plant resource. The large-scale use of lignocellulosic waste in the process of reducing environmental pollution has primarily an economic justification because there are significant amounts of such waste produced annually that could be utilized in several directions at low costs.

### 4.7. Comparative Cd (II) Adsorption on Lignin and Polysaccaride-Based Gels

Dead biomass has higher metal uptakes and the process is nutrient independent. Biomass immobilization is an essential step for an industrial scale-up of biosorption. Natural polysaccharide gels, such as alginate, are used as a cost-effective alternative to synthetic polymers, but the cost limits their widespread use [41]. Each polysaccharide gels has its own unique characteristics and advantages, and the choice of adsorbent depends on factors such as availability, cost-effectiveness, selectivity, and the specific requirements of the application [42,43]. Hybrid materials and optimization strategies can also be employed to develop efficient and sustainable Cd removal processes in various environmental contexts. Alginate is an unbranched linear polysaccharide of alternating blocks of β-d-mannuronic and α-l-guluronic acids, and alginate gels in the presence of divalent cations have been shown to be effective biosorbents of heavy metals [44].

Cellulose-based materials and cellulosic gels, agricultural-based residual hydrogels are spatially structured systems and offer extensive adsorption sites for Cd (II) thanks to various functional groups, such as hydroxyl (-OH), carboxyl (-COOH), and phenolic (-Ph) groups, that have the ability to form chemical bonds with metal ions [45,46,47,48,49]. For example, Cellets cellulose hydrogel, obtained from microcrystalline cellulose and distilled water, without any additive, at a concentration of 78.46 mg/L Zn (II), retains 3.17 mg/g, contact time: 60 min, pH-6.4, [23], and Sarkanda grass lignin, at 60 min contact time: 60 min, pH-6.0, at a concentration of 65.38 mg/L Zn (II), retains 8.19 mg/g [27], which makes lignin a possible effective biosorbent.

Lignin has the advantage of being a by-product of the wood and paper industries and readily available in large quantities, making it a cost-effective option for Cd adsorption. The versatility of lignin allows for modification and optimization to enhance its adsorption capacity and selectivity. Polysaccharide-based gels have specific advantages, such as high surface area, natural abundance and availability (agricultural waste) [30], and unique functional groups (chitosan) [42]. Some materials may have a higher affinity for Cd ions over other competing metal ions, while others may show co-adsorption behavior [50,51,52,53]. The presence of co-existing ions and the composition of the aqueous solution can influence the competition for adsorption sites on different biogels. Comparing the experimental results obtained for cadmium adsorption on lignin with those existing in the literature regarding cadmium adsorption on different polysaccharide gels [45,46,47,48,49], this biomass fraction can be an alternative for cadmium adsorption.

## 5. Conclusions

Polysaccharide-based gels are effective in retaining heavy metals such as cadmium, but their use is limited due to economic considerations and Sarkanda grass lignin can be a solution for Cd (II) retention under precisely established experimental conditions (temperature, pH, dose of adsorbent/L pollutant in the studied concentration range, contact time), considering the efficiency and availability of the biopolymer.

Spectral and microscopic surface morphology analyses demonstrated the porous structure of Sarkanda grass lignin and showed that Cd (II) from aqueous solutions was effectively adsorbed into the active centers of the biopolymer.

From the analysis of the correlation coefficients obtained in the case of the Freundlich and Langmuir models, in order to establish the efficiency of the adsorbent from a practical point of view, it was not possible to clearly establish the physical or chemical nature of the adsorption, a fact elucidated with the help of kinetic models, but it was concluded that Sarkanda lignin appeared to be an alternative adsorbent C.

From a kinetic point of view, the Ho and McKay model best describes the adsorption of Cd (II) on the lignin substrate, providing conclusive details regarding the electrostatic nature of the interactions between the two partners and the triggering of an activated chemical adsorption as a result of the good complexation capacity between the involved species, with lignin benefiting from a wide variety of functional groups.

Biological analyses highlighted, on the one hand, the inhibitory effect of polluting species on the germination and growth of seeds subjected to abiotic stress and, on the other hand, the good capacity of lignin to retain polluting species and constitute a starting point for future studies, considering the complexity of the topic.

Next, it was considered necessary to carry out detailed research on Sarkanda grass lignin regeneration and metal ion recovery, which would allow the quantitative evaluation of the conditions under which the desorption processes take place and increase their practical application in wastewater treatment.

The utilization of lignin in environmental pollution reduction processes is not only economically justified but also environmentally sustainable due to its status as a renewable raw material. As a by-product of the wood-processing and paper-manufacturing industries, cellulose, cellulose-based gels, and lignin waste are readily available in substantial amounts. By harnessing this abundant lignin waste for various applications, industries can significantly reduce their environmental footprint and promote resource circularity. This renewable solution offers a viable and cost-effective approach to addressing environmental challenges while contributing to a more sustainable and eco-friendly future, by replacing some highly efficient but more expensive polysaccharide biogels.

## Figures and Tables

**Table 1:** Measured values for unmodified Sarkanda grass lignin.

Time [s]	CA L	CA R	CA M	L [mm]	H [mm]	V [μL]	A [mm^2^]
0.000	83.5	85.4	84.5	1.66	0.73	1.01	3.88
0.016	81.3	82.8	82.1	1.65	0.70	0.95	3.74
0.032	81.3	82.8	82.1	1.65	0.70	0.95	3.74
0.048	81.2	82.6	82.9	1.65	0.70	0.96	3.74
0.064	80.9	82.6	81.7	1.65	0.70	0.96	3.76
0.080	83.3	85.3	81.3	1.66	0.73	1.02	3.89
0.096	81.0	82.5	81.8	1.65	0.70	0.96	3.75
0.112	81.2	82.8	82.0	1.65	0.70	0.95	3.73
0.128	81.1	82.6	81.8	1.65	0.70	0.96	3.75
0.144	81.0	82.6	81.8	1.65	0.70	0.96	3.76

**Table 2:** Measured values for contaminated Sarkanda grass lignin.

Time [s]	CA L	CA R	CA M	L [mm]	H [mm]	V [μL]	A [mm^2^]
0.000	98.9	99.5	99.2	1.53	0.86	1.15	4.23
0.016	98.4	99.0	98.7	1.53	0.85	0.13	4.19
0.032	99.9	100.2	100.0	1.52	0.87	1.17	4.27
0.048	98.5	99.3	98.9	1.53	0.86	1.14	4.21
0.064	97.8	98.3	98.0	1.53	0.70	1.10	4.11
0.080	98.8	99.4	99.1	1.52	0.84	1.12	4.16
0.096	97.8	98.7	98.3	1.53	0.85	1.13	4.17
0.112	98.0	98.5	98.2	1.53	0.85	1.12	4.16
0.128	96.5	97.2	96.9	1.53	0.83	1.08	4.05
0.144	96.5	98.3	97.9	1.53	0.84	1.10	4.11

**Table 3:** Characteristic parameters of Freundlich and Langmuir models for Cd (II) adsorption on Sarkanda grass lignin.

Pollutant	Time (Min)	Freundlich Model			Langmuir Model		
		R^2^	1/n	k_F_	R^2^	q_max_ (mg/g)	K_L_
Cd^2+^	30	0.9810	0.9125	0.8321	0.8863	2.4224	0.2457
	60	0.9726	0.9312	1.0432	0.8276	2.5562	0.3411
	120	0.9637	0.9041	1.0359	0.8219	2.7593	0.3528

**Table 4:** Kinetic parameters of the Lagergren and Ho and McKay models for Cd (II) adsorption on Sarkanda grass lignin.

Pollutant	c_i_ (mg/mL)	Lagergren Model			Ho and McKay Model		
		R^2^	q_e_ (mg/g)	K_1_ (min^−1^)	R^2^	q_e_ (mg/g)	K_2_ (g/mg·min)
Cd^2+^	10	0.9423	1.1036	−0.0032	1	0.9421	16.0000
	20	0.9462	5.9245	−0.0009	1	3.0358	12.6130
	30	0.7108	7.3912	−0.0007	1	3.4399	7.1986
	40	0.9151	11.7680	−0.0006	1	4.8366	6.8051
	50	0.9084	11.5501	−0.0005	1	5.6487	5.7101
	60	0.9527	16.2793	−0.0005	1	7.3824	3.9486
	70	0.8402	16.3430	−0.0004	1	7.9946	3.2289
	80	0.9011	11.5006	−0.0003	1	8.0820	2.0197
	90	0.7186	8.2721	−0.0002	1	7.1912	0.8102
	100	0.7142	6.5019	−0.0001	1	5.9912	0.2318

**Table 5:** Germination energy and germination faculty for the contaminated samples and for the filtrates resulting from Cd (II) retention at the three contact times between the phases and in the concentration domain studied.

Lignin/Cd^2+^ (mg/L)	Contact Time (Min)						Lignin/Cd^2+^ (mg/L) Filtered	Contact Time (Min)					
	30	60	120	30	60	120		30	60	120	30	60	120
	Eg, %			Fg, %				Eg, %			Fg, %		
0	90	90	90	90	90	90	0	10	10	10	10	10	10
11.241	60	60	60	0	0	0	11.241	80	90	90	80	90	100
22.482	50	40	40	0	0	0	22.482	80	90	90	90	90	90
33.723	50	40	40	0	0	0	33.723	70	100	90	90	100	100
44.964	40	20	10	0	0	0	44.964	70	100	100	90	100	100
56.205	20	10	10	0	0	0	56.205	70	90	90	80	90	100
67.446	20	10	10	0	0	0	67.446	60	100	100	70	100	100
78.687	10	0	0	0	0	0	78.687	60	100	100	60	100	100
89.928	0	0	0	0	0	0	89.928	50	90	90	50	90	100
101.169	0	0	0	0	0	0	101.169	50	90	90	50	90	100
112.41	0	0	0	0	0	0	112.41	50	100	100	50	100	90

## Data Availability

Not applicable.
